# An Assessment of Mental Health Status of Undergraduate Medical Trainees in the University of Calabar, Nigeria: A Cross-Sectional Study

**DOI:** 10.3889/oamjms.2015.068

**Published:** 2015-06-10

**Authors:** Afiong Oku, Oboko Oku, Eme Owoaje, Emmanuel Monjok

**Affiliations:** 1 *Department of Community Medicine, University of Calabar, Calabar, Cross River State, Nigeria*; 2 *Department of Anaesthesia, University of Calabar, Calabar, Cross River State, Nigeria*; 3 *Department of Community Medicine, University of Ibadan, Oyo State, Nigeria*

**Keywords:** Mental health, medical students, Calabar, Nigeria, General health questionnaire 12

## Abstract

**BACKGROUND:**

The mental health status of medical students has been proven to be poor compared to their peers in other disciplines and has led to grave personal and professional consequences. This subject has however remained largely unexplored in our medical school.

**AIM:**

The study was therefore conducted to assess the prevalence of mental health of medical students in the University of Calabar, Cross river state, Nigeria.

**METHODOLOGY:**

A descriptive cross-sectional survey of 451 randomly selected medical students from the pre-clinical and clinical levels of study in the University of Calabar. A self administered questionnaire including the GHQ12 was used to elicit information from the respondents. A score of ≥ 3 suggested poor mental while a score < 3 represented good mental health. Data were summarized using proportions, and χ2 test was used to explore associations between categorical variables. Level of significance was set at p < 0.05.

**RESULTS:**

The mean age of the respondents was 23.4 ± 4.3 years, 63.8% were males, 34.8% were from the preclinical and 65.2% from clinical levels of study. Based on the GHQ categorisation, 39.2% had a poor mental health status, compared to 60.8% with good mental health status. The factors significantly associated with poor mental health, were recent experience of mistreatment by trainers or colleagues, perceived inadequate monthly allowance and perception that medical training is stressful (p < 0.05).

**CONCLUSION:**

With more than a third of undergraduate medical trainees with traits of poor mental health, provision of accessible mental health services/counselling is strongly recommended early in their training.

## Introduction

Mental health is defined as a state of well-being in which every individual realizes his or her own potential, can cope with the normal stresses of life, can work productively and fruitfully, and is able to make a contribution to her or his community [1]. A person with positive mental health uses interpersonal assets and skills to function successfully in his or her daily life. Mental health problems emerge when these assets and skills begin to deteriorate, resulting in a struggle to cope with life’s challenges and responsibility.

Mental disorders account for a large proportion of the disease burden in young people in all societies. Most mental disorders begin during youth (12—24 years of age), although they are often first detected later in life. Poor mental health is strongly related to other health and development concerns in young people notably lower educational achievements, substance abuse, violence, and poor reproductive and sexual health [2]. Research has proven that the mental health of medical students in particular, worsens from the beginning of medical school and remains poor throughout training [3-6]. On a personal level this has been found to contribute to substance abuse, broken relationship, suicide and attrition while on a professional level studies suggest that can contribute to cynicism and subsequently may affect students care of patient relationship with faculty and ultimately the culture of medical profession [7] Medical students’ wellbeing as a precursor to physicians’ wellbeing represents a critical aspect of the training, as stable healthy and resilient physicians are better equipped for emotionally and physically demanding tasks of providing care comfort and hope to patients.

Tertiary medical training across the globe has been regarded as being highly stressful. Dahlin et al rightly observed that promoting and nurturing wellbeing during medical school, equipping graduates with the skills necessary to recognize personal distress, (to determine when they need to seek assistance) and to develop strategies to promote their own wellbeing is fundamental to promoting professionalism [8]. Unfortunately, some aspects of the training process have unintended negative consequences on students ‘personal health. The mental health of medical students has attracted particular concerns because of their future potential impact on patients and the entire population. Although a doctor with psychological morbidity traits and poor mental health is much more likely to present a risk to himself, the spectre of potential harm also extends to patients, medical school is profoundly difficult time for physicians in training. It is physically and emotionally demanding and thus may produce stress at levels which are hazardous to the physical and psychological wellbeing of the students. However, medical practice demands the highest standards of performance and conduct, thus assessment of future medical practitioners becomes imperative. At the start of medical school, medical students have mental health similar to their nonmedical peers [9, 10]. Unfortunately, numerous studies suggest that students’ mental health worsens during the course of undergraduate medical training [4, 5, 11-13]. Mental disorders might appear early or, even during the years of medical training [14]. A Swedish study of medical students found the prevalence of depressive symptoms to be 12.9%, which was significantly higher than that in the general population [8]. Prevalence of psychiatric morbidity was 26% in Australian final year medical students, which increased significantly during internship [15]. Similarly, an increase in the prevalence of mental health problems requiring treatment was noted in Norwegian medical students but there was no increase in help-seeking [16]. In a study from the United Kingdom, more than one third of first-year students had poor mental health when measured with the General Health Questionnaire 12, (GHQ 12) which assessed anxiety and depression [17]. Another study from the United Kingdom of the same year students found that the incidence of poor mental health on the General Health Questionnaire 12 doubled during the first year, increasing from 25% to 52% [11]. A similar study conducted in Nepal India revealed that 20.9% of medical students interviewed had traits of poor mental health status [18]. In Nigeria, Omigbodun and colleagues reported that the medical/dental students had a higher GHQ scores compared to nursing and physiotherapy students [19].

There is a global awareness on the negative consequences of excessive stress encountered in the course of medical training which ultimately affects the physician’s wellbeing and quality of care given to the patients. Poor mental health status has also been shown to affect academic performance and future options for affected students [7, 13, 20, 21]. Several reports have been documented on stress/psychological wellbeing among medical students in developed countries, but few studies in the south western part of the Nigeria [19, 22]. However, only one study has been conducted in the south-south region of the country. Therefore, there is an urgent need for medical educators to understand the causes of student’s distress, potential adverse personal and professional consequences and institutional factors that can positively or negatively influence student’s health which is what this study would reveal. This study would provide insight into the psychosocial problems these students face in the course of their training.

The aim of this study therefore was to assess the mental health status of the medical students in the University of Calabar, Nigeria.

## Materials and Methods

The study was carried out among medical students at the College of Medicine, University of Calabar Cross River state. Nigeria. The College of Medicine was established in 1975. It is one of the largest universities in south- south zone of Nigeria and enrolled students represent a wide range of socioeconomic levels among the general population. The institution on the average produces an average of 80 medical doctors annually. In their first year, the students are exposed to orientation courses thereafter admitted into the medical school proper (Pre-clinical levels) where they offer paramedical courses in their second and third year which include anatomy, physiology and biochemistry, thereafter they move over to the clinical years in where they are exposed to clinical courses. The academic calendar of the medical students is usually longer and usually different from the other departments in the University. Most often, when others are on holidays they have to forfeit theirs in order to complete their voluminous syllabus. Majority, of the students reside in the hostels with a few residing off campus. The Pre-clinical students, (levels 2-3) are usually accommodated with other students in the general hostels which are usually overcrowded with 10-12 occupants per room. The fourth to sixth year (clinical) students are usually accommodated in hostels near the Teaching Hospital where most of their clinical rotations occur. Recreational activities provided for the students are minimal and most often underutilized by medical students. They include; Table tennis, chess, draft (indoor games) and a football pitch.

Students are mainly from the south -south and south west region of the country. It is located in Calabar South Local government area (LGA) of Cross River state. In 2010 there were 917 medical students spanning through all the six levels of study.

### Study population

Medical students in the University of Calabar Medical School drawn from all levels of study.

### Study design

Descriptive cross-sectional survey was used.

### Sample size calculation

The Kish and Leslie Sample size formula for descriptive study was used to calculate the sample size:

n= z^2^pq/d^2^

n= minimum sample size; z = critical value at 95% confidence interval; p = proportion of medical students who identified hostel accommodation as major stressor = 43.8 % [19]; d= level of precision taken as 0.05; q= 1-p = 0.6; z = 1.96; p = 0.44.

Therefore

n = 1.96 ^2^*0.44*0.56/0.05 *0.05

= + 10% allowance for non response

= 378.8/0.9 = 409.8

= 420.8

The minimum sample size calculated is approximately 425. For Populations less than 10,000 n = n

1+n/N

N = 425/1+425/917

= 291.1

The sample size was approximated to 300.

### Eligibility criteria

**Inclusion criteria:** All consenting medical students currently undergoing training in the University of Calabar were eligible to participate.

**Exclusion criteria:** Non-consenting and students who were not in school when the study was carried out were excluded from the study.

### Sampling technique

The number of participants per class was derived using proportionate to size method. This was done as follows:

A total number of medical students per class was obtained from the office of the College Secretary, in all there were a total number of 917medical students.

At the time the study was carried out, the total number of medical students was 681. The reason being that only four classes were available levels (1, 2, 4 and 5). The third level students had just written their professional examination and were on vacation and the final year students had just graduated from the medical school so the sample size was adjusted to the remaining classes available A sample fraction of 300681 was applied to each class e.g. for year 1 there was a total of 130 students. The desired sample for Year 1 = 130 *300/681 = 57 this was done for the rest of the classes. For the 2^nd^ year class = 126*300/681 =56.

**Table:** 

Level of study	Desired sample per class	Number studied
1	130 *300/681	57
2	126*300/681	56
4	297*300/681	131
5	128*300/681	56
**Total**	**57+56+131+56**	**300**

A sample fraction of 300/681 was applied to each class e.g. for year 1 there was a total of 130 students.

Thereafter, the respondents were randomly selected using Probability sampling (simple random sampling). Balloting was the method employed in selecting respondents per class into the study

The list of all the students was obtained from each class representative who constituted the sample frame. After meeting with the class representatives, of each class, dates were decided by them when the study was conducted. After their lectures, the purpose of the study was explained to the students by the Researcher after which the students were asked to remain seated. For example, in the First year class of 130 students, a basket containing envelopes with 90 Yes’ and the remaining No’s was passed round and each student expected to pick an envelope. The required number of 57 was increased to ensure a large number of students were included into the study. After the last student had picked from the basket, they were told to open their envelopes. The students who picked Yes were asked to remain seated while the No’s asked to leave the lecture venue. Questionnaires were then distributed to those seated after careful instructions given by the Lead researcher. The exercise was then replicated in the other available classes. The minimum sample size was increased to 451.

Data were collected using a pretested semi structured self administered questionnaire which consisted of the following sections: Socio-demographic characteristics of the respondents and assessment of psychological wellbeing the General Health Questionnaire 12 (GHQ 12).

### Data management

Questionnaires were inspected daily so as to detect errors, and omissions to ensure that it is properly filled. Questionnaires were manually sorted out, coded before entry and cleaned. Thereafter the data were entered into a computer for statistical analysis using Statistical Package for the Social Sciences (SPSS) version 16.0. Frequency, proportions, means and standard deviation was generated to summarize variables. Chi square tests were used to test associations between categorical variables. Level of significance was kept at 5%. The GHQ is a self-administered questionnaire designed to detect psychiatric disorders in community and other settings such as primary care. The GHQ-12 was chosen because it has been validated for use in this environment and is short and easy to complete, containing only 12 items. The standard GHQ method of scoring 0–0–1–1 for each item was employed, which allows a maximum score of 12. A cut-off point of 3 for GHQ 12 has been used successfully in this environment and those with a GHQ score of 3 above suggest poor mental health status.

### Ethical consideration

Ethical approval was sought for and obtained from the University Of Calabar Teaching Hospital Ethical Review Committee before data collection commenced. Careful explanation of the purpose, content and implication was made known to the participants. Informed consent done in writing was obtained from each respondent after careful explanation of study objectives has been made. Confidentiality was assured as names were not required from the subjects. Respondents and non respondents were not penalized for participating or not participating in the study.

## Results

The mean age of respondents was 23.44 ± 4.38 years. Majority, 288 (63.8%) of the respondents interviewed were males, 437 (96.9%) were single and 294 (65.2%) of the students were from the clinical level of study. About half, 233 (51.7%) of the medical students interviewed resided on campus and all were of the Christian religious faith. More students, 167 (37.7%) had a monthly allowance of between N10, 000 < N20,000. However, majority, 325 (72.1%) reported that their monthly allowance was inadequate (Table 1).

**Table 1:** Socio-demographic characteristics (n = 451)

Variable	Frequency n (%)
**Age (years)**	
≤ 19	80 (17.7)
20-24	200 (62.1)
25-29	134 (29.7)
≥30	37 (8.2)
**Mean ±SD**	23.44 ± 4.38
**Gender**	
Male	288 (63.8)
Female	163 (36.2)
**Marital status**	
Single	437 (96.9)
Married	14 (3.1)
**Residence**	
On campus	233 (51.7)
Off campus	218 (48.3)
**Level of study**	
Year 1	82 (18.2)
Year 2 Year	75 (16.6)
Year 4	184 (40.8)
Year 5	110 (24.4)
**Monthly allowance**	
≤ N 9999	125 (27.7)
N 10000-19999	167 (37.0)
N 20000- 29999	75 (16.6)
N30000-39999	26 (5.8)
≥ N40000	24 (5.3)
No response	34 (7.3)
**Median (range)**	**N 12,000 (500-100,000)**
**Adequacy of monthly allowance**	
Adequate	123 (27.3)
Not adequate	325 (72.1)
Nil	3 (0.6)

Almost all 425 (94.2%) perceived their undergraduate training as stressful, more than a third, 160 (35.5%) had recently experienced mistreatment and only 86 (19.1%) indicated that they would not read medicine if given a second chance (Table 2).

**Table 2:** Perceived stress, mistreatment and second chance at medicine

Variable	Frequency n (%)
	
**Perceived medical school as stressful**	
Yes	425 (94.2)
No	26 (5.8)
**Recent experience of mistreatment or abuse**	
Yes	160 (35.5)
No	291 (64.5)
**Will study Medicine if given a second chance**	
Yes	365 (80.9)
No	86 (19.1)

The responses of the General Health Questionnaire by the respondents are shown in Table 3. The mean score attained was 2.35 + 2.29. Most of the responses of the GHQ were positive by majority of the students except losing much sleep over worry 343 (76.1%), feeling of being unable to overcome difficulties 378 (83.8) and felt constantly under strain 267 (59.2%). In addition, majority 407 (90.2) lost confidence in themselves, 431 (95.6%), thought of themselves as worthless and 354 (78.5%) felt unhappy and depressed. On the other hand most respondents reportedly faced up to their problems 400 (88.7%), majority, felt reasonably happy 376 (83.4%) and 292 (64.7%) indicated that they enjoyed their daily activity all things being equal.

**Table 3:** Responses of the General health Questionnaire (GHQ 12) N= 451

Variable	Frequency (%)
**Concentrated in what you were doing**	
Yes	316 (70.1)
No	135 (29.9)
**Lost much sleep over worry**	
Yes	343 (76.1)
No	108 (23.9)
**Felt you were playing a useful part in things**	
Yes	381 (84.5)
No	70 (15.5)
**Felt capable of making decisions about things**	
Yes	407 (90.2)
No	44 (9.8)
**Felt constantly under strain**	
Yes	267 (59.2)
No	184 (40.8)
**Felt you couldn’t overcome difficulties**	
Yes	378 (83.8)
No	73 (16.2)
**Enjoyed a normal day to day activity**	
Yes	292 (64.7)
No	159 (35.3)
** Faced up to problems**	
Yes	400 (88.7)
No	51 (11.3)
**Felt unhappy and depressed**	
Yes	354 (78.5)
No	97 (21.5)
**Lost confidence in self**	
Yes	407 (90.2)
No	44 (9.8)
**Thought of yourself as a worthless person**	
Yes	431 (95.6)
No	20 (4.4)
**Felt reasonably happy all things considered**	
Yes	376 (83.4)
No	75 (16.6)

**Figure 1:**
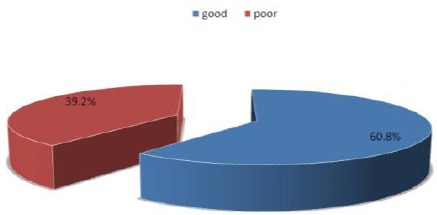
*Mental health status of respondents*.

Assessment of mental health (Figure 1) of medical students using the GHQ12 revealed that 39.2% of respondents had traits of poor mental health.

The association between the socio-demographic characteristics/other variables and mental health status (Table 4) revealed no statistical significant association. On the other hand, perceiving monthly allowance as inadequate, medical school as stressful, experienced mistreatment and refused to take up a career in Medicine if given a second chance.

**Table 4:** Association between some socio-demographic variables and mental health status

Variable	Mental health status		χ^2^	p-value
	Poor (%)	Good (%)		
**Age**				
<19	35(42.7)	47(57.3)		
20-24	78(40.4)	115(59.6)		
25-29	45(35.4)	82(64.6)	1.97	0.578
≥30	12(32.4)	25(67.6)		
**Sex**				
Male	116(59.9)	172(40.1)		
Female	62(38.0)	101(62.0)	0.12	0.671
**Marital status**				
Single	174(39.8)	263(60.2)		
Married	3(21.4)	11(78.6)	1.92	0.165
**Residence**				
On campus	98(42.2)	134(57.8)		
Off campus	78(35.9)	139(64.1)	1.87	0.17
**Level of study**				
Year 1	33(40.2)	49(59.8)		
Year 2	31(41.3)	44(58.7)		
Year 4	72(39.1)	112(60.9)	0.35	0.95
Year 5	41(37.3)	69(62.7)		
**Perceived monthly allowance as sufficient**				
**Adequate**				
Yes	39(31.7)	84(68.3)		
No	137(42.2)	188(57.8)	4.08	0.043*
**Monthly allowance**				
≤N 12000	15(44.1)	19(55.9)		
> N 12000	87(41.0)	12(59.0)	1.23	0.54
**Second chance at medicine**				
Yes	132(36.2)	233(63.8)	7.62	0.006*
No	45(52.3)	41(47.7)		
**Perceived medical training as stressful**				
Yes	172(40.5)	253(59.5)		
No	5(19.2)	21(80.8)	4.64	0.031*
**Experienced mistreatment**				
Yes	74(46.3)	86(53.7)	5.10	0.024*
No	103(35.4)	188(64.6)		

* statistically significant.

## Discussion

The mental health status of medical trainees has long been recognised as a cause for concern in both developed and developing countries [15, 16, 19, 22].

The mean age of the respondents was consistent with similar studies carried out in medical schools in Nigeria [19, 22, 23], in which the mean age was 23.71 ± 3.55 and majority of the students were between the ages 20-24 years. Overall, more than three out of five of the respondents were males, this is quite different from what was obtained in a study conducted in Malaysia which reported with a higher female to male ratio 62.3% were female medical students and 37.7% males [24]. This goes to show that in Nigeria the medical profession is still a purely a male dominated one, though there has been increasing number of females entering into the profession. This study also revealed that majority of the medical students were drawn from the fourth level of study, the reason behind this was that at the time of the study the students in the third level of study had just passed their professional examination so there were two fourth year classes.

Majority (96.9%) were single compared to only 3.1% who were married this is similar to reports from other studies [22]. Christianity was the dominant religion amongst the students. This could be attributable to the fact that most students came from the southern part of the country which comprises of mainly Christians compared to the Northern part of Nigeria.

### Mental health status of respondents

Assessment of psychological morbidity or mental health status of the respondents using the GHQ12 was a key finding in this study. The prevalence of psychological morbidity was 39.2%. This was found to be high compared to studies reported from both High income and Low and middle income countries [4, 10, 14, 17, 25]. Earlier studies from United Kingdom using a cut off score of 3– 4 reported slightly lower findings for prevalence of psychiatric morbidity [4, 17, 26]. Furthermore, varying estimates of psychiatric distress were demonstrated in other studies not using the same cut-off as this study. In a study from India using GHQ 12 and a cut off score of 4–5 suggested an overall prevalence of psychological morbidity of 20.9% [18]. Reports from Malaysia and Turkey using a cut off of 4 had a prevalence of 46.2%, and 47.9%, respectively. These figures are higher than what was observed in the present study. However, studies conducted among medical students in Pakistan and Spain which used the same cut off as the present study showed comparable results as observed in present study [27, 28]. In Nigeria using the same cut off lower rates were observed in south western Nigeria, in both clinical and preclinical students a cumulative prevalence of 32% for both preclinical and clinical levels of study [22]. The reasons for our high levels of psychological morbidity are likely to be complex and cannot be attributed to single issues and to be rationalized by the perceived medical school stress and our highly stressful educational environment in which the medical students are. Personal characteristics of our students themselves and possible previous mental health problems may also be considered. These variations in mental health status of medical students shows that effective supportive and mental health services still need to be instituted as a necessary part of the under graduate medical training both in developed and developing countries.

The socio-demographic characteristics of our sample did not significantly influence the risk of having poor states of mental health. Neither gender nor any of the other socio demographic variables in our study was found to have a significant relationship with psychiatric morbidity. This finding was consistent with other studies [20, 21, 29, 30]. The reason behind this remains unclear. However, other studies reported contrary results which indicated associations with these variables [18].

Significant associations were observed among students who perceived their monthly allowance as inadequate and having traits of poor mental health. This finding is consistent with other studies [22, 29, 31]. Other factors which negatively influenced the students mental health status included recent mistreatment, were more than two out of every five respondent who experienced recent mistreatment or abuse were more likely to have traits of poor mental health status. This finding was in contrary to that of Shoukat and colleagues who reported no significant association between mistreatment and psychological morbidity [28]. Likewise the perception of medical training as stressful was also significantly associated with poor mental health states. These findings were consistent with what was observed in Pakistan [28].

The cross-sectional nature of this study was reflective of a period of time and would not allow for results to be generalized for the medical undergraduate population. The classes included in the study were those available at the time the study was being carried out. The absence of the third and final level students was a major limitation.

A major finding was the level of psychological morbidity or mental health status of the medical students interviewed. The proportion of medical students identified as having traits of poor mental health using the GHQ 12 was quite high and is a cause for concern for our future medical professionals. The presence of accessible mental health services to help identify these students with psychological morbidity early would help to address most of the problems students encountered.
